# Supplementary material for: Integrating human activity into food environments can better predict cardiometabolic diseases in the United States
Source: Nat Commun. 2023 Nov 13;14:7326. doi: 10.1038/s41467-023-42667-8 (PMC10643374; doi:10.1038/s41467-023-42667-8)
Supplement: Supplementary file 1 — Supplementary Information [file 41467_2023_42667_MOESM1_ESM.pdf]

Supplementary Information

Supplementary Table 1. Number of business establishments in 2018–2019 SafeGraph and 2021 NAICS by category

Category	SafeGraph	NAICS	Total visits in SafeGraph
Supermarket	85,910	86,178	306,848,201
Convenience store	52,865	55,559	142,982,105
Fruits and vegetable market	10,525	7,375	11,923,905
Limited-service restaurant	208,675	275,209	945,029,126
Warehouse club	1,390	1,839	67,754,033

Supplementary Table 2. Summary statistics of the variables included in this study

Variable	# Observations	Mean	SD	Min	Max
RFAI	73,315	27.946	8.634	0.000	91.304
mRFEI	71,684	11.62	10.902	0.000	100.000
SafeGraph-based location food environment index	71,834	30.555	21.582	0.000	100.000
Percent food-related visits within ½ mile boundary of the home census tract	73,602	0.208	0.139	0.000	1.000
Median distance traveled (log-transformed)	73,602	1.492	0.798	-2.321	7.177
Percentile ranking of SVI theme 1 (socioeconomic status)	72,173	0.500	0.289	0.000	1.000
Percentile ranking of SVI theme 2 (household composition & disability)	72,274	0.500	0.289	0.000	1.000
Percentile ranking of SVI theme 3 (minority status & language)	72,378	0.500	0.289	0.000	1.000
Percentile ranking of SVI theme 4 (housing type & transportation)	72,201	0.500	0.289	0.000	1.000
Urban indicator	72,493	0.761	0.427	0.000	1.000
Food desert indicator	72,493	0.279	0.449	0.000	1.000
High blood pressure prevalence	70,336	32.492	7.362	5.100	72.300
Coronary heart disease prevalence	70,336	6.115	2.059	0.300	37.100
Diabetes prevalence	70,336	10.963	3.727	0.700	44.200
Obesity prevalence	70,336	32.629	6.824	11.900	58.300
High cholesterol prevalence	70,336	31.832	4.794	6.200	52.900
Percent minority	72,335	0.371	0.313	0.000	1.000
Percent low income	72,335	0.339	0.212	0.000	1.000
Percent less than high school education	72,335	0.132	0.120	0.000	1.000
Percent under age 5	72,335	0.059	0.039	0.000	0.511
Percent over age 64	72,335	0.160	0.100	0.000	1.000
Percent female	73,298	0.508	0.048	0.000	1.000
Non-Hispanic White community indicator	73,298	0.669	0.471	0.000	1.000
Hispanic community indicator	73,298	0.108	0.310	0.000	1.000
Non-Hispanic Black community indicator	73,298	0.082	0.275	0.000	1.000
Per capita visits to food retailers during 2018–2019 (log-transformed)	73,264	1.346	0.548	-6.034	8.626
Food retailers to population ratio (log-transformed)	71,508	-5.754	1.039	-9.455	5.689

Census tract population (log-transformed)	73,298	8.279	0.548	0.693	11.185
Population density (people per square mile; log-transformed)	73,296	7.137	2.157	-4.230	12.431

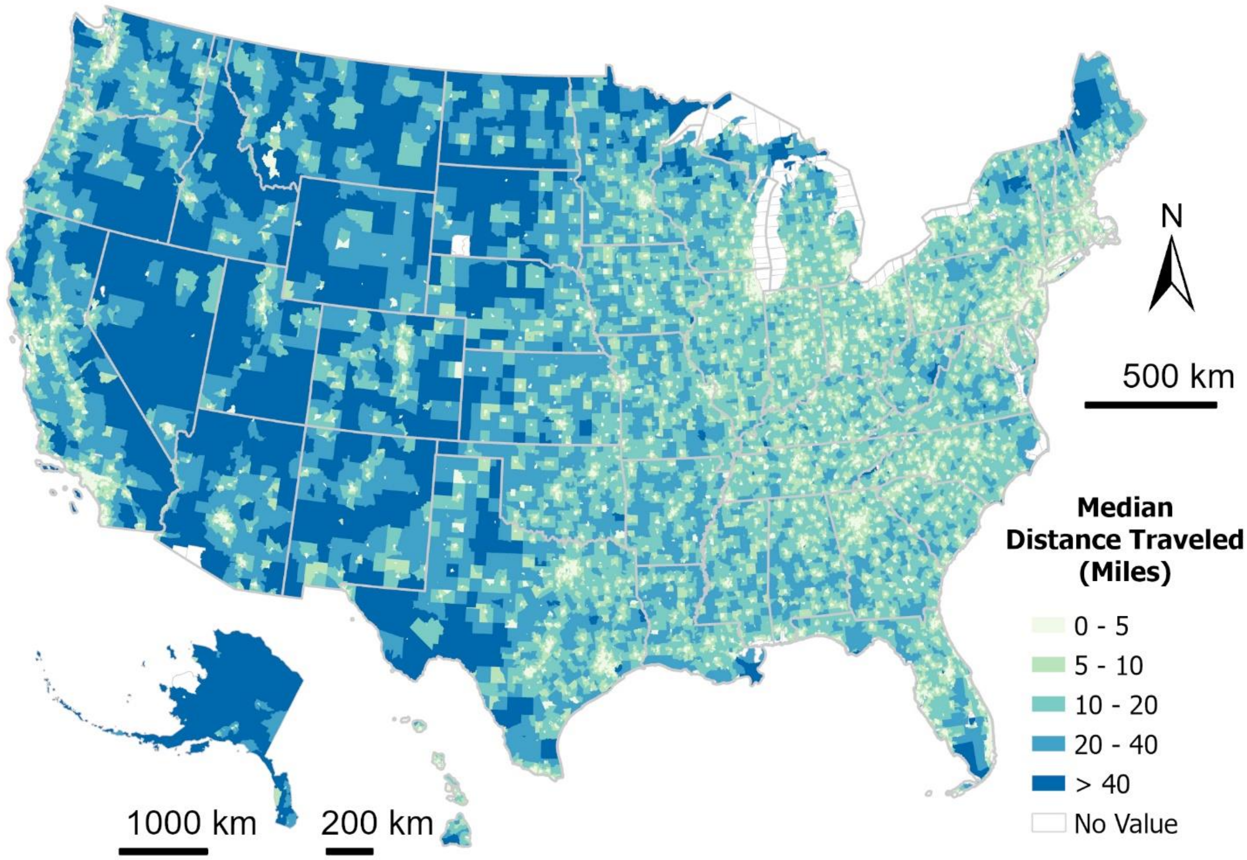

Supplementary Figure 1. US census tract level map for the median distance traveled to food retailers in 2018–2019. The map was made in ESRI ArcGIS Pro 3.0.2.

Supplementary Table 3. Regression results of the association between sociodemographic factors and median distance traveled to food retailers. In different specifications, we used (A) percentile ranking of SVI theme 3 (minority status & language) as the independent variable; (B) indicators of census tracts with predominantly non-Hispanic White, non-Hispanic Black, and Hispanic populations as the independent variables. Two-sided t-tests were used to test all coefficients. *n* = 72,139 census tracts.

	Linear regression		Quantile regression	
	Coefficient (95% CI)	P value	Coefficient (95% CI)	P value
Percentile ranking of SVI theme 1 (socioeconomic status)	-0.053 (-0.071, -0.034)	<.00001	0.360 (0.222, 0.498)	<.00001
Percentile ranking of SVI theme 2 (household composition & disability)	-0.055 (-0.070, -0.041)	<.00001	-0.515 (-0.624, -0.406)	<.00001
Percentile ranking of SVI theme 3 (minority status & language)	0.249 (0.234, 0.264)	<.00001	1.351 (1.238, 1.464)	<.00001
Percentile ranking of SVI theme 4 (housing type & transportation)	-0.164 (-0.177, -0.151)	<.00001	-0.347 (-0.447, -0.246)	<.00001
Food desert indicator	-0.039 (-0.048, -0.030)	<.00001	-0.357 (-0.426, -0.290)	<.00001
Urban indicator	-0.255 (-0.269, -0.242)	<.00001	-3.967 (-4.064, -3.869)	<.00001
Per capita visits to food retailers during 2018–2019 (log-transformed)	-0.122 (-0.130, -0.115)	<.00001	-0.786 (-0.841, -0.732)	<.00001
Population density (log-transformed)	-0.276 (-0.280, -0.274)	<.00001	-1.249 (-1.269, -1.229)	<.00001

(A)

	Linear regression		Quantile Regression	
	Coefficient (95% CI)	P value	Coefficient (95% CI)	P value
Percentile ranking of SVI theme 1 (socioeconomic Status)	-0.069 (-0.088, -0.051)	<.00001	0.296 (0.153, 0.440)	.00005
Percentile ranking of SVI theme 2 (household composition & disability)	-0.083 (-0.098, -0.068)	<.00001	-0.635 (-0.746, -0.524)	<.00001

Indicator for predominantly non-Hispanic White census tract	-0.150 (-0.161, -0.140)	<.00001	-0.650 (-0.728, -0.571)	<.00001
Indicator for predominantly Hispanic census tract	-0.015 (-0.029, -0.002)	0.02203	0.076 (-0.027, 0.180)	0.14884
Indicator for predominantly non-Hispanic Black census tract	0.035 (0.021, 0.049)	<.00001	0.173 (0.062, 0.282)	0.00218
Percentile ranking of SVI theme 4 (housing type & transportation)	-0.135 (-0.148, -0.121)	<.00001	-0.215 (-0.317, -0.114)	0.00003
Food desert indicator	-0.046 (-0.055, -0.037)	<.00001	-0.382 (-0.451, -0.313)	<.00001
Urban indicator	-0.244 (-0.257, -0.231)	<.00001	-3.915 (-4.014, -3.816)	<.00001
Per capita visits to food retailers during 2018-2019 (log-transformed)	-0.113 (-0.121, -0.106)	<.00001	-0.736 (-0.791, -0.681)	<.00001
Population density (log-transformed)	-0.274 (-0.276, -0.271)	<.00001	-1.211 (-1.231, -1.190)	<.00001

(B)

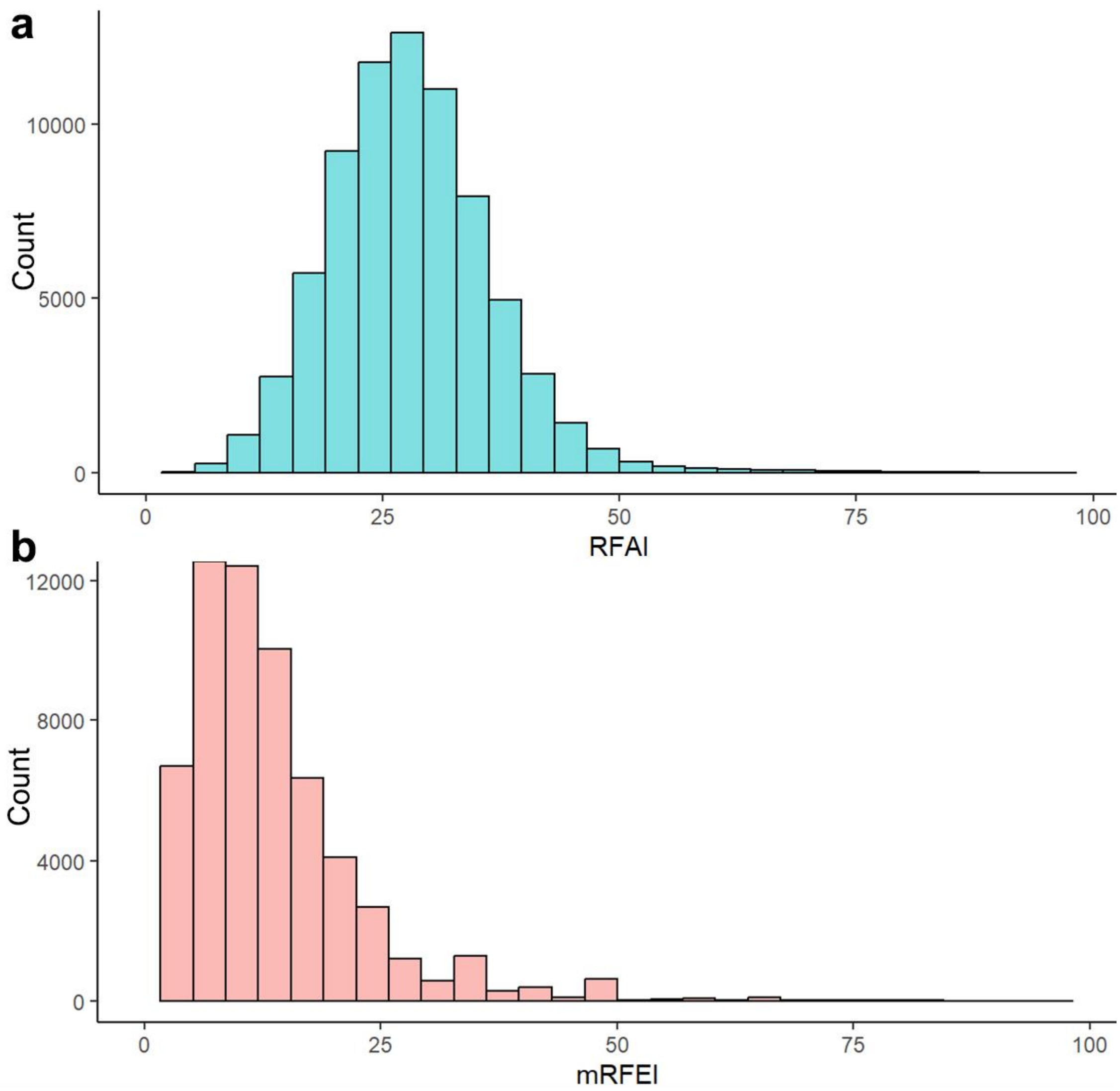

Supplementary Figure 2. Distribution of (a) RFAI (n = 73,315 census tracts) and (b) mRFEI (n = 71,684 census tracts) at the census tract level.

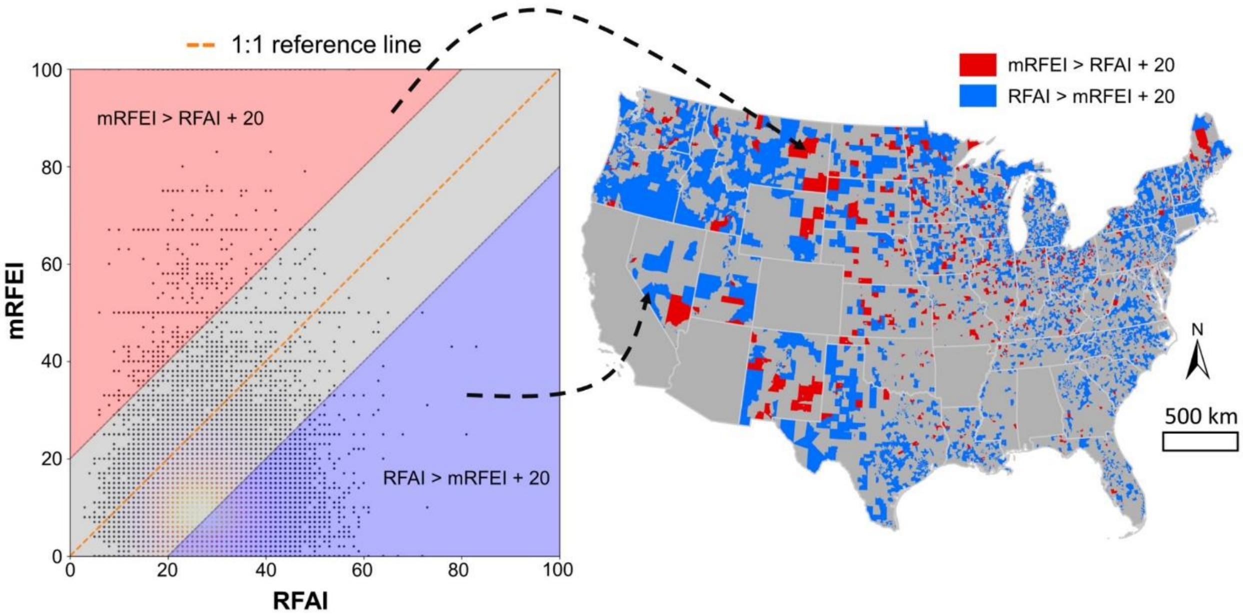

Supplementary Figure 3. Scatterplot of RFAI and mRFEI, and the mismatch between the two on the census tract level. Blue regions represent that RFAI is higher than mRFEI and red regions represent that mRFEI is higher than RFAI. The map was made in ESRI ArcGIS Pro 3.0.2.

Supplementary Table 4. Regression results of the association between sociodemographic factors and RFAI as well as mRFEI. In different specifications, we used (A) percentile ranking of SVI theme 3 (minority status & language) as the independent variable; (B) indicators of census tracts with predominantly non-Hispanic White, non-Hispanic Black, and Hispanic populations as the independent variables. Two-sided t-tests were used to test all coefficients. *n* = 72,124 census tracts for RFAI and *n* =71,520 census tracts for mRFEI.

	RFAI		mRFEI	
	Coefficient (95% CI)	P value	Coefficient (95% CI)	P value
Percentile ranking of SVI theme 1 (socioeconomic status)	-5.814 (-6.107, -5.521)	<.00001	-3.927 (-4.376, -3.478)	<.00001
Percentile ranking of SVI theme 2 (household composition & disability)	0.647 (0.415, 0.877)	<.00001	1.637 (1.283, 1.991)	<.00001
Percentile ranking of SVI theme 3 (minority status & language)	5.579 (5.339, 5.819)	<.00001	-0.034 (-0.403, 0.334)	0.85677
Percentile ranking of SVI theme 4 (housing type & transportation)	-2.317 (-2.530, -2.104)	<.00001	0.326 (0.001, 0.652)	0.04932
Food desert indicator	-1.499 (-1.644, -1.355)	<.00001	-1.263 (-1.484, -1.042)	<.00001
Urban indicator	-0.646 (-0.853, -0.438)	<.00001	0.472 (0.154, 0.789)	0.00364
Per capita visits to food retailers during 2018–2019 (log-transformed)	-7.591 (-7.707, -7.474)	<.00001	-2.312 (-2.490, -2.134)	<.00001
Population density (log-transformed)	0.569 (0.526, 0.611)	<.00001	-0.778 (-0.844, -0.711)	<.00001

(A)

	RFAI		mRFEI	
	Coefficient (95% CI)	P value	Coefficient (95% CI)	P value
Percentile ranking of SVI theme 1 (socioeconomic status)	-5.658 (-5.961, -5.357)	<.00001	-3.704 (-4.164, -3.244)	<.00001
Percentile ranking of SVI theme 2 (household	0.046 (-0.185, 0.277)	0.69523	1.952 (1.599, 2.305)	<.00001

composition & disability)				
Indicator for predominantly non-Hispanic White census tract	-1.199 (-1.363, -1.035)	<.00001	0.309 (0.060, 0.559)	0.01497
Indicator for predominantly Hispanic census tract	2.992 (2.773, 3.209)	<.00001	1.166 (0.834, 1.497)	<.00001
Indicator for predominantly non-Hispanic Black census tract	0.463 (0.231, 0.694)	0.00009	-1.919 (-2.271, -1.567)	<.00001
Percentile ranking of SVI theme 4 (housing type & transportation)	-1.694 (-1.907, -1.481)	<.00001	0.121 (-0.203, 0.446)	0.46180
Food desert indicator	-1.496 (-1.641, -1.351)	<.00001	-1.147 (-1.368, -0.926)	<.00001
Urban indicator	-0.333 (-0.541, -0.126)	0.00163	0.517 (0.201, 0.834)	0.00137
Per capita visits to food retailers during 2018–2019 (log-transformed)	-7.420 (-7.537, -7.304)	<.00001	-2.363 (-2.540, -2.185)	<.00001
Population density (log-transformed)	0.693 (0.651, 0.734)	<.00001	-0.757 (-0.822, -0.692)	<.00001

(B)

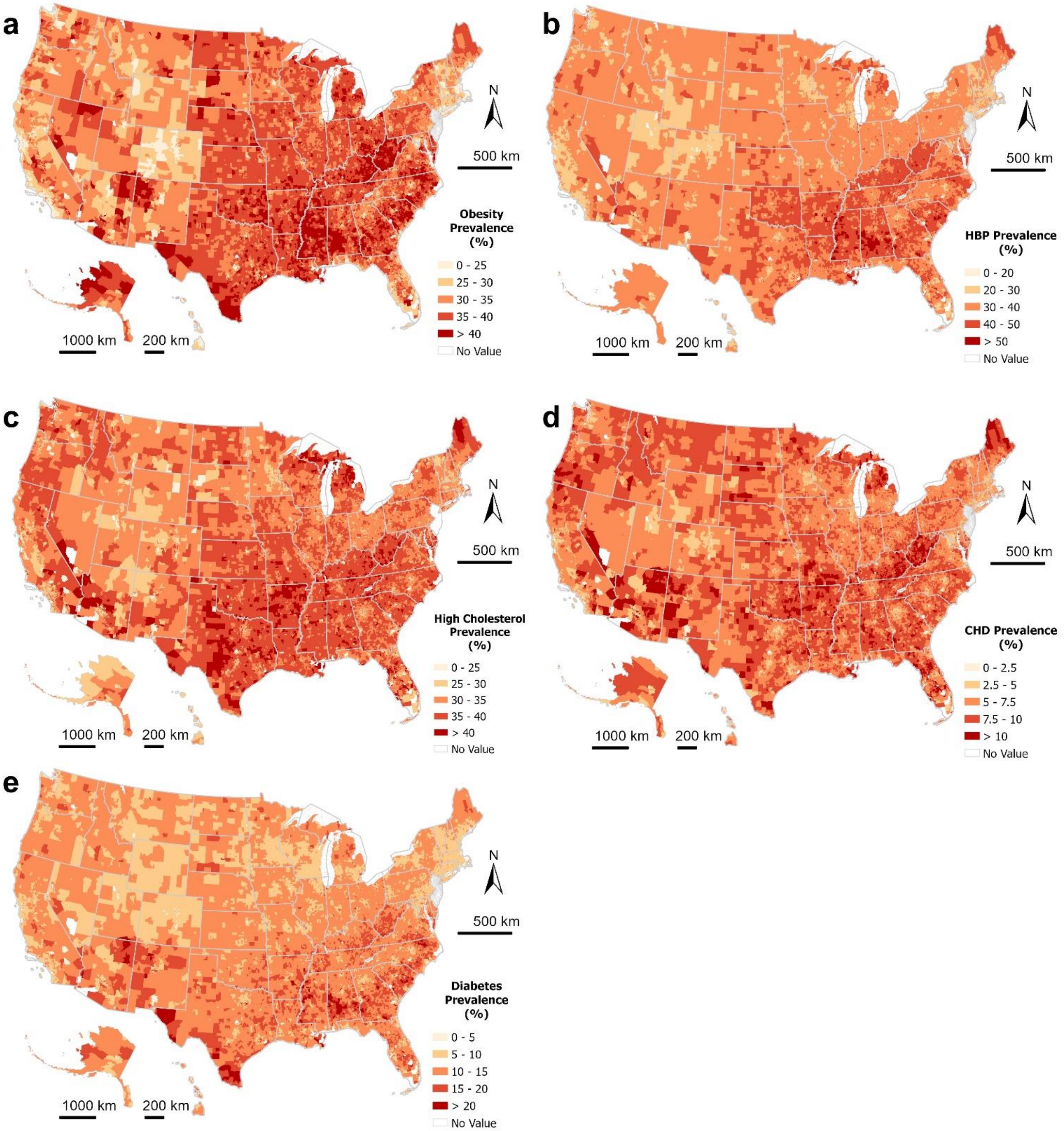

Supplementary Figure 4. Census tract level prevalence of (A) obesity among adults aged ≥ 18, (B) high blood pressure among adults aged ≥ 18, (C) high cholesterol among adults aged ≥ 18, (D) coronary heart disease among adults aged ≥ 18, and (E) diagnosed diabetes among adults aged ≥ 18. The maps were made in ESRI ArcGIS Pro 3.0.2.

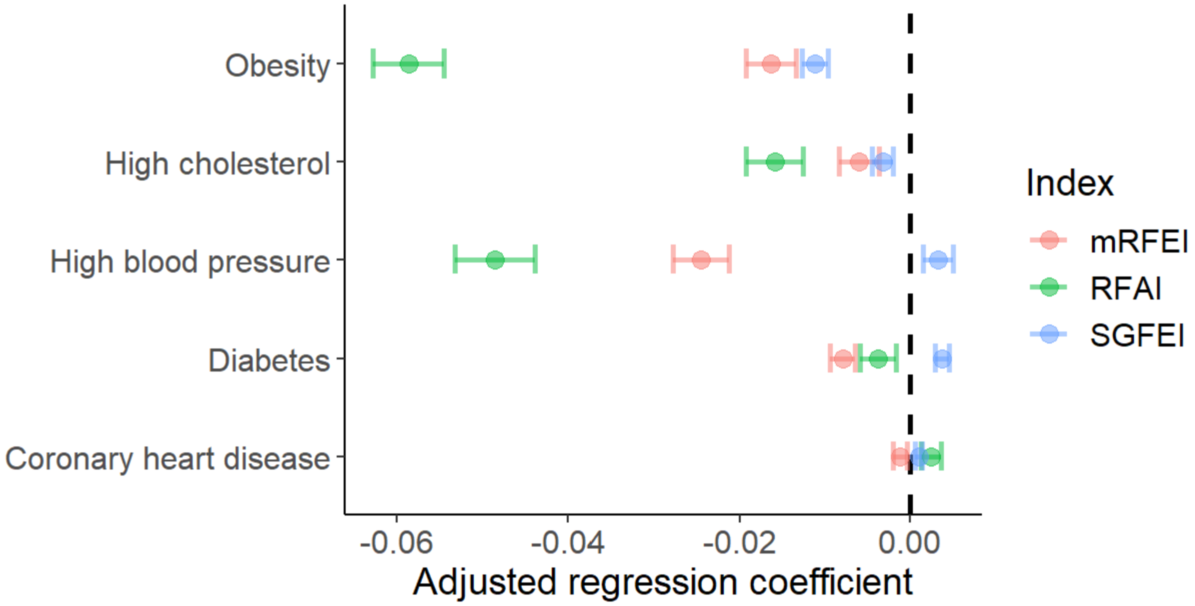

Supplementary Figure 5. Independent association between cardiometabolic disease prevalence and each index at the census tract level. SafeGraph food environment index (SGFEI), a location-based food environment index, was created based on all food retailers included in the 2018–2019 SafeGraph data using the following formula $SGFEI = \frac{\#\ Healthy\ Food\ Retailers}{\#\ Healthy\ Food\ Retailers + \#\ Less\ Healthy\ Food\ Retailers}$ (see methods for the definition of healthy and unhealthy food retailers). All models controlled for the percentile ranking of four SVI themes (i.e., socioeconomic status, household composition and disability, minority status, housing type and transportation), food desert and urban indicators, per capita food retailer visits in 2018–2019 (log-transformed), percent of the population that are female, minority, low income, have less than a high school education, are under age 5 or over age 64, median family income, and total number of food retailers (log-transformed) in each census tract. Center of the error bar represents the regression coefficient and the error bar represents 95% CI. *n* = 67,856 census tracts for mRFEI, *n* = 68,260 census tracts for RFAI, and *n* = 68,262 census tracts for SGFEI.

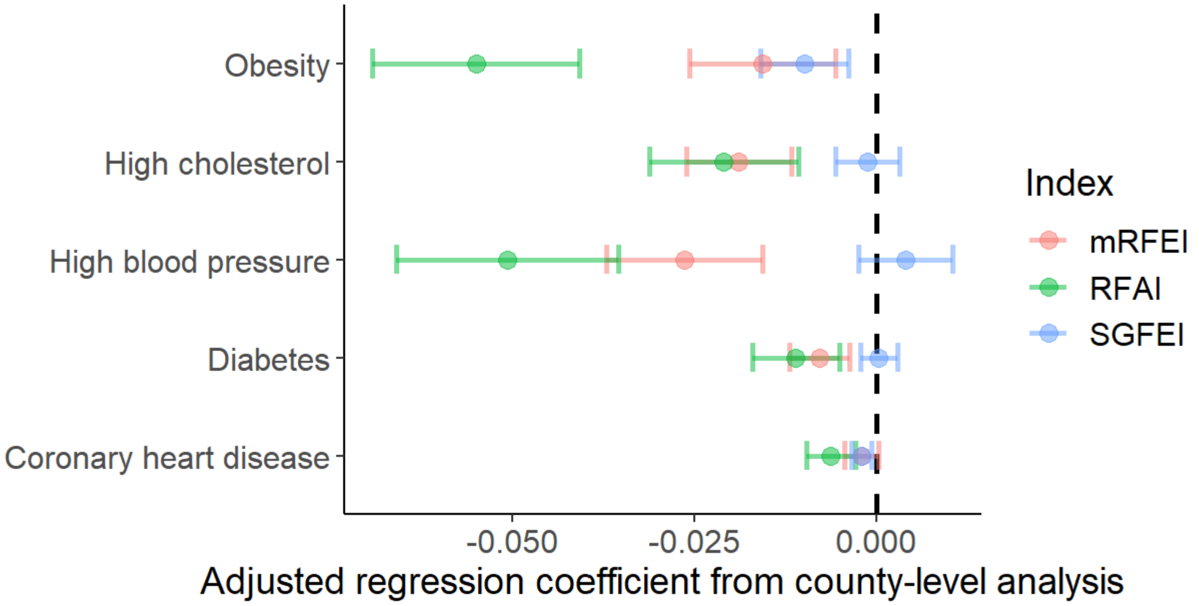

Supplementary Figure 6. Independent association between cardiometabolic disease prevalence and each index using county-level analysis. All models controlled for the percentile ranking of four SVI themes (i.e., socioeconomic status, household composition and disability, minority status, housing type and transportation), food desert and urban indicators, per capita food retailer visits in 2018–2019 (log-transformed), percent of the population that are female, minority, low income, have less than a high school education, are under age 5 or over age 64, median family income, and total number of food retailers (log-transformed) in each county. We recreated RFAI and SGFEI, total number of food retailers, and per capita food retailer visits in 2018–2019 at the county level, and we created all other measures by averaging (weighted by census population) measures for all census tracts within each county. Center of the error bar represents the regression coefficient and the error bar represents 95% CI. *n* = 3,105 counties for mRFEI, *n* = 3,115 counties for RFAI, and *n* = 3,091 counties for SGFEI.

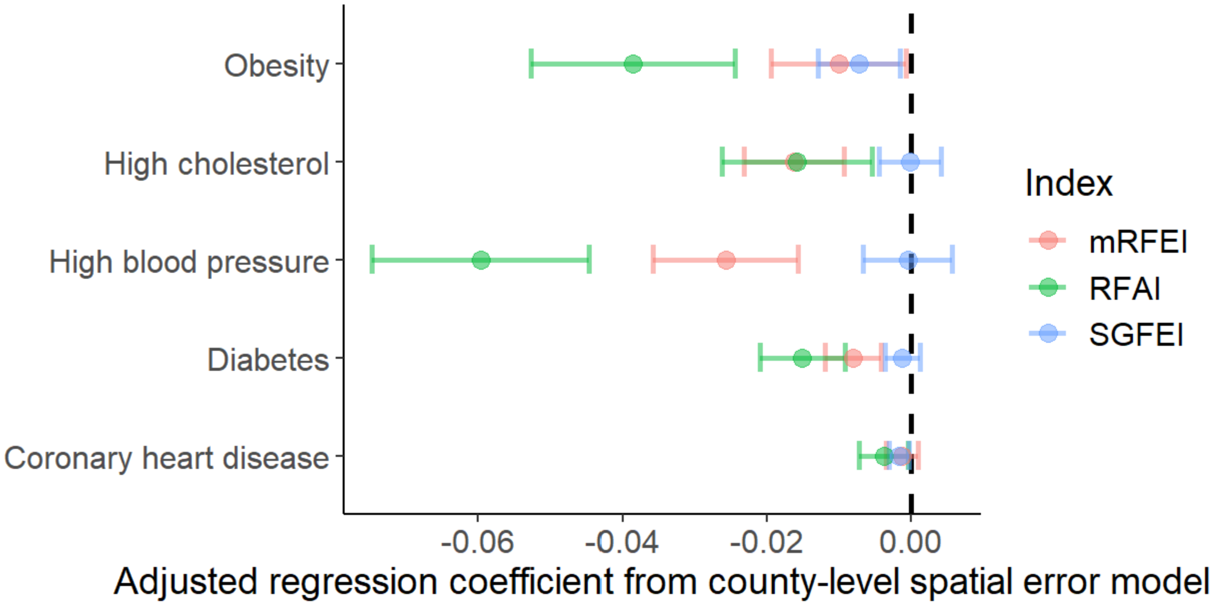

Supplementary Figure 7. Independent association between cardiometabolic disease prevalence and each index using county-level spatial error model. Spatial weights were based on inverse distance with spectral normalization. All models controlled for the percentile ranking of four SVI themes (i.e., socioeconomic status, household composition and disability, minority status, housing type and transportation), food desert and urban indicators, per capita food retailer visits in 2018–2019 (log-transformed), percent of the population that are female, minority, low income, have less than a high school education, are under age 5 or over age 64, median family income, and total number of food retailers (log-transformed) in each county. Center of the error bar represents the regression coefficient and the error bar represents 95% CI. *n* = 3,105 counties for mRFEI, *n* = 3,115 counties for RFAI, and *n* = 3,091 counties for SGFEI.

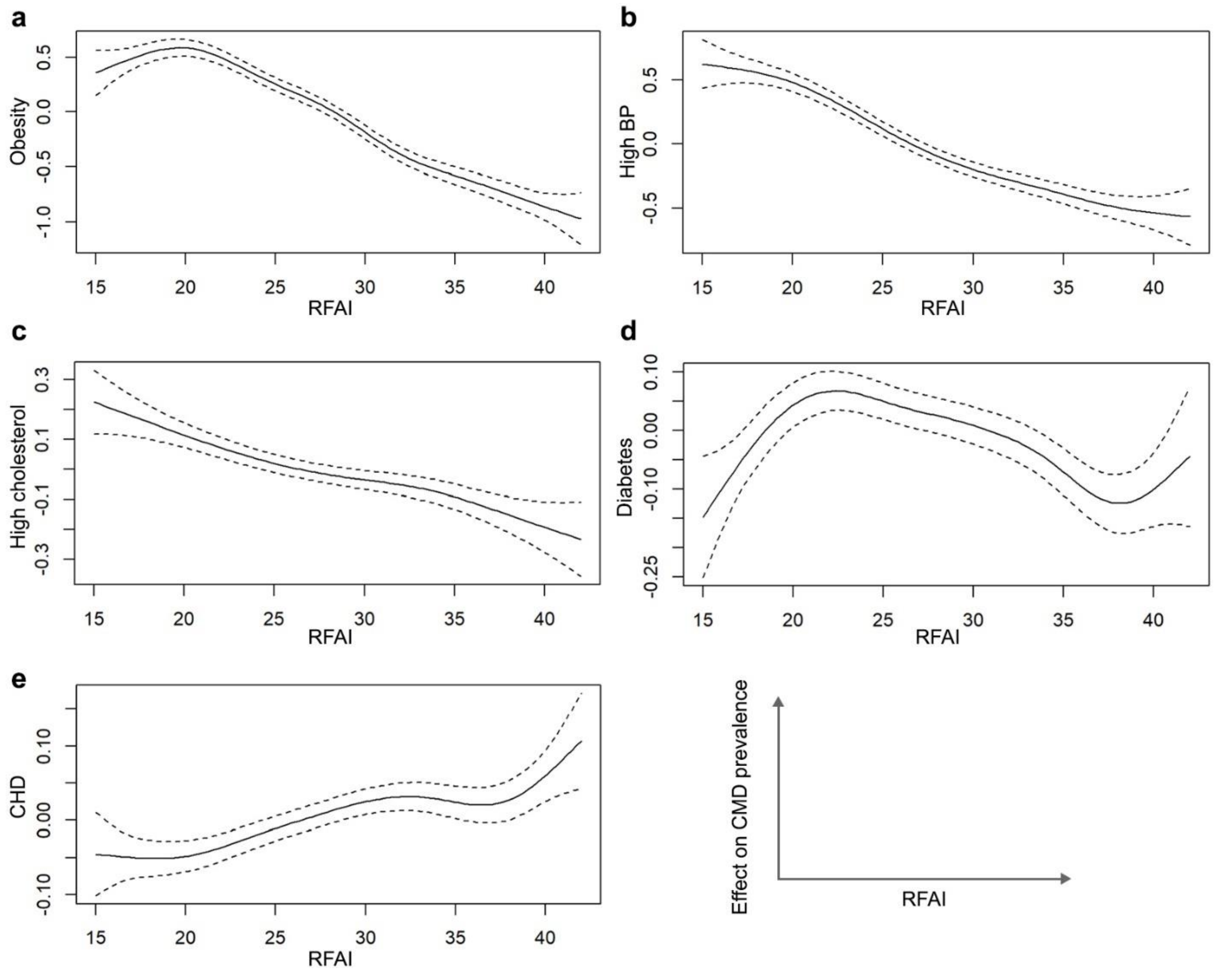

Supplementary Figure 8. Non-linear effect of RFAI (with 95% CI) on the prevalence of (a) obesity, (b) high blood pressure, (c) high cholesterol, (d) diabetes and (e) coronary heart disease. Here RFAI ranges from 15 (5th percentile) to 42 (95th percentile). We performed the analyses using generalized additive model framework, all models controlled for the percentile ranking of four SVI themes (i.e., socioeconomic status, household composition and disability, minority status, housing type and transportation), food desert and urban indicators, per capita food retailer visits in 2018–2019 (log-transformed), percent of the population that are female, minority, low income, have less than a high school education, are under age 5 or over age 64, median family income, and total number of food retailers (log-transformed) in each census tract. *n* = 62,144 census tracts.

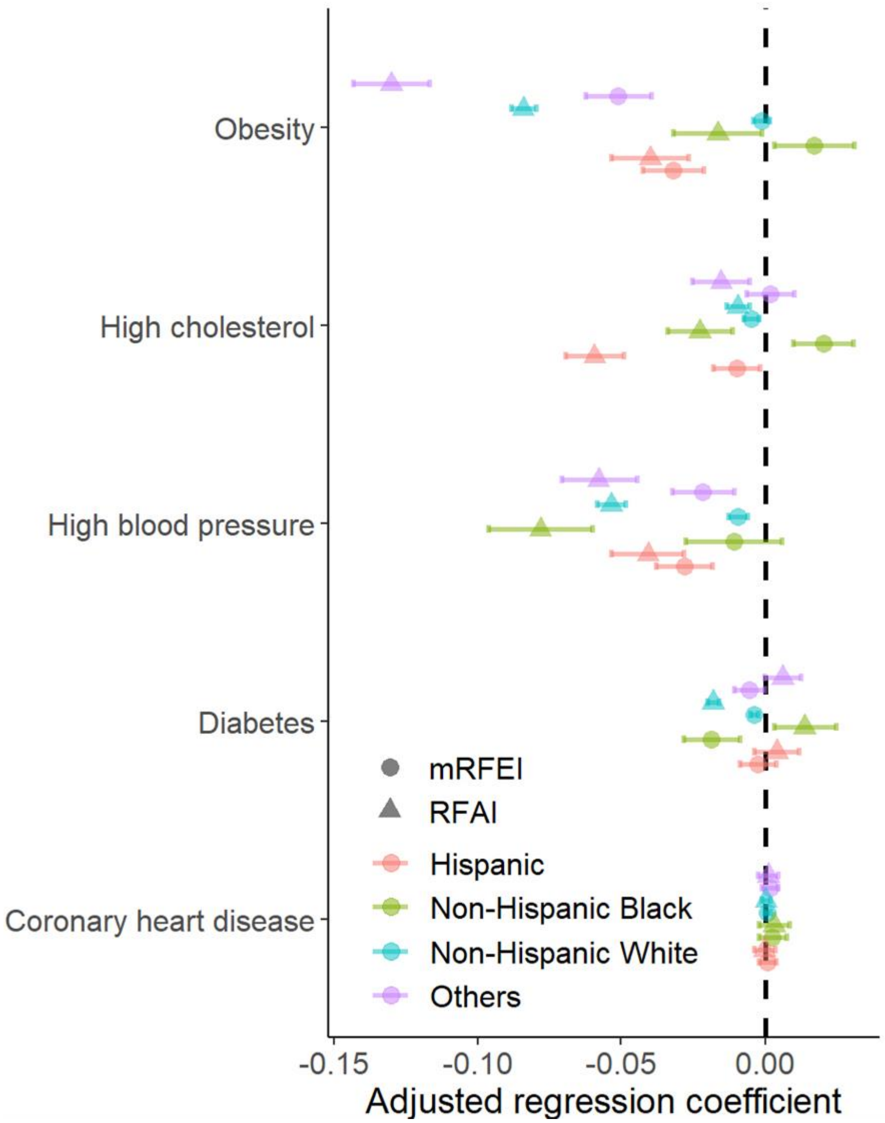

Supplementary Figure 9. Independent association between cardiometabolic disease prevalence and each index by racial/ethnic group. We performed the analysis for census tracts with each predominant racial/ethnic group separately, and all models controlled for the percentile ranking of three SVI themes (i.e., socioeconomic status, household composition and disability, housing type and transportation), food desert and urban indicators, per capita food retailer visits in 2018–2019 (log-transformed), percent of the population that are female, low income, less than high school education, under age 5 or over age 64, median family income, and total number of food retailers (log-transformed) in each census tract. Center of the error bar represents the regression coefficient and the error bar represents 95% CI. For mRFEI, *n* = 6,679 census tracts for Hispanic, *n* = 5,727 census tracts for Non-Hispanic Black, *n* = 45,876 census tracts for Non-Hispanic White, and *n* = 9,574 census tracts for others. For RFAI, *n* = 6,688 census tracts for Hispanic, *n* = 5,732 census tracts for Non-Hispanic Black, *n* = 46,238 census tracts for Non-Hispanic White, and *n* = 9,602 census tracts for others.
