# Supplementary material for: Integrating human activity into food environments can better predict cardiometabolic diseases in the United States
Source: Nat Commun. 2023 Nov 13;14:7326. doi: 10.1038/s41467-023-42667-8 (PMC10643374; doi:10.1038/s41467-023-42667-8)
Supplement: Supplementary file 3 — Reporting Summary [file 41467_2023_42667_MOESM3_ESM.pdf]

nature portfolio

Corresponding author(s): DAPR NCOMMS-23-15926A

Last updated by author(s): Oct 5, 2023

Reporting Summary

Nature Portfolio wishes to improve the reproducibility of the work that we publish. This form provides structure for consistency and transparency in reporting. For further information on Nature Portfolio policies, see our Editorial Policies and the Editorial Policy Checklist.

Statistics

For all statistical analyses, confirm that the following items are present in the figure legend, table legend, main text, or Methods section.

n/a	Confirmed	
☐	☒	The exact sample size (*n*) for each experimental group/condition, given as a discrete number and unit of measurement
☐	☒	A statement on whether measurements were taken from distinct samples or whether the same sample was measured repeatedly
☐	☒	The statistical test(s) used AND whether they are one- or two-sided<br>*Only common tests should be described solely by name; describe more complex techniques in the Methods section.*
☐	☒	A description of all covariates tested
☐	☒	A description of any assumptions or corrections, such as tests of normality and adjustment for multiple comparisons
☐	☒	A full description of the statistical parameters including central tendency (e.g. means) or other basic estimates (e.g. regression coefficient) AND variation (e.g. standard deviation) or associated estimates of uncertainty (e.g. confidence intervals)
☐	☒	For null hypothesis testing, the test statistic (e.g. *F*, *t*, *r*) with confidence intervals, effect sizes, degrees of freedom and *P* value noted<br>*Give P values as exact values whenever suitable.*
☒	☐	For Bayesian analysis, information on the choice of priors and Markov chain Monte Carlo settings
☒	☐	For hierarchical and complex designs, identification of the appropriate level for tests and full reporting of outcomes
☒	☐	Estimates of effect sizes (e.g. Cohen's *d*, Pearson's *r*), indicating how they were calculated

Our web collection on statistics for biologists contains articles on many of the points above.

Software and code

Policy information about availability of computer code

Data collection	no software was used in this study as this is a secondary data analysis and all data were publicly available online
Data analysis	STATA 17.0, R 4.2.2 and R package "ggplot2" 3.4.3, ESRI ArcGIS Pro 3.0.2 were used to analyze the data and visualize the results. Code used in this study are available at https://dataverse.harvard.edu/dataset.xhtml?persistentId=doi:10.7910/DVN/DMJDVL

For manuscripts utilizing custom algorithms or software that are central to the research but not yet described in published literature, software must be made available to editors and reviewers. We strongly encourage code deposition in a community repository (e.g. GitHub). See the Nature Portfolio guidelines for submitting code & software for further information.

Data

Policy information about availability of data

All manuscripts must include a data availability statement. This statement should provide the following information, where applicable:

- Accession codes, unique identifiers, or web links for publicly available datasets
- A description of any restrictions on data availability
- For clinical datasets or third party data, please ensure that the statement adheres to our policy

data used in this study are available at https://dataverse.harvard.edu/dataset.xhtml?persistentId=doi:10.7910/DVN/DMJDVL

Research involving human participants, their data, or biological material

Policy information about studies with human participants or human data. See also policy information about sex, gender (identity/presentation), and sexual orientation and race, ethnicity and racism.

Reporting on sex and gender	N/A
Reporting on race, ethnicity, or other socially relevant groupings	N/A
Population characteristics	N/A
Recruitment	N/A
Ethics oversight	N/A

Note that full information on the approval of the study protocol must also be provided in the manuscript.

Field-specific reporting

Please select the one below that is the best fit for your research. If you are not sure, read the appropriate sections before making your selection.

☐ Life sciences ☒ Behavioural & social sciences ☐ Ecological, evolutionary & environmental sciences

For a reference copy of the document with all sections, see nature.com/documents/nr-reporting-summary-flat.pdf

Behavioural & social sciences study design

All studies must disclose on these points even when the disclosure is negative.

Study description	This is an observational study and the data was quantitative at the census tract level
Research sample	We used existing dataset from multiple sources, including modified retail food environment index (mREFI) in 2011 from the Centers for Disease Control and Prevention's (CDC) Division of Nutrition, Physical Activity, and Obesity, anonymized aggregated GPS data in 2018-2019 from SafeGraph's Core Places and Patterns datasets, Cardiometabolic related disease prevalence in 2019 from PLACES data provided by CDC's Division of Population Health, Epidemiology and Surveillance Branch, and census tracts' demographic and socioeconomic characteristics from CDC (2018 SVI), 5-year (2013–2018) American Community Survey and 2020 decennial census.
Sampling strategy	This is a US nationwide census tract level study and we obtained the aforementioned data on all census tracts in the US.
Data collection	Our study is a secondary data analysis and all data used in this study were previously collected by third parties. GPS-tracking data was collected by SafeGraph through tracking GPS-enabled smartphones, mRFEI was collected by CDC from business information, all other data were collected through surveys and interviews. All data were de-identified and aggregated on the census tract level. SafeGraph's Core Places and Patterns datasets are commercially available, all other data are free and publicly available. Researchers were blinded to any experimental condition or study hypotheses.
Timing	modified retail food environment index (mREFI) was based on business information up to April 2011. Anonymized aggregated GPS data was collected from January 2018 to December 2019. Cardiometabolic related disease prevalence was based on Behavioral Risk Factor Surveillance System (BRFSS) data collected in 2019. Census tracts' demographic and socioeconomic characteristics were based on three data sources collected during 2013–2018 and 2020. See research sample and manuscript for more details.
Data exclusions	no data were excluded from the analyses
Non-participation	not applicable as all data used are secondary and on the census tract level.
Randomization	This is an observational study, in the analysis we controlled for multiple demographic and socioeconomic characteristics in each model. See details in the methods section

Reporting for specific materials, systems and methods

We require information from authors about some types of materials, experimental systems and methods used in many studies. Here, indicate whether each material, system or method listed is relevant to your study. If you are not sure if a list item applies to your research, read the appropriate section before selecting a response.

Materials & experimental systems

n/a	Involved in the study
☒	☐ Antibodies
☒	☐ Eukaryotic cell lines
☒	☐ Palaeontology and archaeology
☒	☐ Animals and other organisms
☒	☐ Clinical data
☒	☐ Dual use research of concern
☒	☐ Plants

Methods

n/a	Involved in the study
☒	☐ ChIP-seq
☒	☐ Flow cytometry
☒	☐ MRI-based neuroimaging
